# SARS-CoV-2 Spike Protein XBB.1.5 Mutations Altered Four Conserved Antigenic Determinants

**DOI:** 10.3390/ijms27041940

**Published:** 2026-02-18

**Authors:** Ekrem Akbulut, Meltem Yildirim, Huseyin Kahraman

**Affiliations:** 1 Department of Bioengineering, Malatya Turgut Ozal University, 44900 Malatya, Türkiye; 2 Department of Biomedical Engineering, Malatya Turgut Ozal University, 44900 Malatya, Türkiye; 3 Department of Biology, Inonu University, 44280 Malatya, Türkiye

**Keywords:** SARS-CoV-2, COVID-19, spike, XBB.1.5, epitope, immune escape

## Abstract

The continuous evolution of SARS-CoV-2 affects its infectivity and ability to evade the immune system. The XBB.1.5 subvariant carries numerous mutations compared to previous Omicron variants and exhibits significant evasion of polyclonal neutralizing antibodies. In this study, the mechanistic effects of mutations in the XBB.1.5 spike protein on structural stability, antigenic markers, and antibody epitopes were analyzed using homology modeling, epitope prediction, protein stability analysis, coarse-grained dynamic simulations, and chain-specific interface mapping. Thirty-eight amino acid substitutions were identified relative to Wuhan-Hu-1, including 22 in the receptor-binding region. The prefusion trimeric fold was conserved, with localized rearrangements in the N-terminal domain, receptor-binding domain, and S1/S2 region. Linear B-cell epitope prediction yielded similar epitope counts and length distributions in wild-type and XBB.1.5, but only moderate residue-level overlap (Jaccard ≈ 0.40–0.62), indicating epitope turnover and alteration of four conserved antigenic determinants. Functional screening suggested that ~45% of substitutions could affect protein function. Chain-specific interface analysis of the A–B protomer interface indicated preserved inter-protomer coupling with modest repacking of the polar/directional contacts. Overall, XBB.1.5 appears to maintain ACE2 engagement while redistributing antibody targets, underscoring the need for updated vaccine formulations and therapeutic antibodies.

## 1. Introduction

Coronavirus disease 2019 (COVID-19), the etiological agent of which is caused by severe acute respiratory syndrome coronavirus-2 (SARS-CoV-2), is one of the most significant health problems of the twenty-first century. SARS-CoV-2, which has caused the death of more than 7 million people, is a positive-sense single-stranded RNA virus. The spike (S) protein, essential for the infectivity of SARS-CoV-2 and other coronaviruses, belongs to class I viral fusion proteins, similar to several proteins that have been extensively studied, such as HIV’s Gp41 and the influenza virus hemagglutinin. Like other fusogens in this category, the S-protein is trimeric and features a large ectodomain visible on the virion surface, an -helical transmembrane domain (TMD), and a small endodomain. The S protein is an important drug and vaccine target because it activates cell entry by binding to the angiotensin-converting enzyme-2 (ACE2) receptor during viral invasion [1,2,3,4]. Changes resulting from the continuous evolution of the SARS-CoV-2 genome have resulted in structural changes in the S protein. These structural changes have significant implications for SARS-CoV-2’s infectivity, immune evasion, and pathogenicity [5]. The physiological activities of the S protein make it a primary target for the development of neutralizing antibodies and vaccines.

After receptor binding and proteolytic activation, spikes undergo major conformational rearrangements, in which S1 dissociates and S2 refolds to mediate membrane fusion via the conserved HR1/HR2 six-helix bundle (6-HB). The HR1 “fusion core” is central to 6-HB stabilization and is thus a plausible site where mutations could modulate fusion efficiency. Consistent with this, Oliva et al. (2021) reported recurrent substitutions in the HR1 fusion core (S929T, D936Y, S939F) and proposed that D936Y can weaken stabilizing interactions in the post-fusion assembly (via loss of an inter-monomer salt-bridge network), increasing flexibility [6]. Together, these observations suggest that spike evolution may tune not only S1-mediated receptor engagement/immune escape but also the downstream fusion machinery, providing a context for interpreting variant-specific effects.

To date, five variants of concern (VOCs) have been identified: Alpha, Beta, Gamma, Delta, and Omicron. Among these, Omicron exhibits the greatest divergence from the original Wuhan-Hu-1. The omicron variant and its sublineages are characterized by a high density of mutations in the S protein, particularly in the receptor-binding domain (RBD) and the N-terminal domain (NTD). This allows for the evasion of vaccines and therapeutic monoclonal antibodies while preserving effective ACE2 binding [7,8]. XBB, a lineage derived from two BA.2 sublineages (BJ.1 and BM.1.1.1), contains extensive receptor-binding domain (RBD) substitutions associated with antibody resistance. The XBB.1.5 sublineage differs from the parental gene, XBB.1, by the RBD mutation F486P in the receptor-binding motif [9].

Epidemiological and experimental evidence has demonstrated that XBB.1.5 possesses a substantial growth advantage over co-circulating variants, attributable to improved ACE2 binding and marked evasion of polyclonal neutralizing antibodies elicited by vaccination, infection, or hybrid immunity. Neutralization studies have shown markedly reduced serum neutralizing titers against XBB and XBB.1.5 across various immune systems, including during BA.5 breakthrough infections and after multiple vaccine doses [10,11,12]. This highlights the necessity of understanding the structural and antigenic foundations of this phenotype and revising vaccine formulations accordingly.

In this context, in silico approaches offer a powerful, rapid, and cost-effective strategy to study how complex mutations shape protein stability, epitope presentation, and conformational dynamics in the S protein [13]. The integration of homology modeling, epitope prediction, stability prediction, and coarse-grained dynamics can generate mechanistic hypotheses that complement experimental structural biology and neutralization data and aid in the prioritization of epitopes and regions for rational immunogen and antibody design [14,15].

This study systematically analyzed, using a comprehensive methodology, the changes in S protein structure, stability, and epitope presentation resulting from mutations observed in the XBB.1.5 variant of SARS-CoV-2. The resulting structural framework elucidates how the combined effects of multiple mutations in XBB.1.5 can predispose the virus to increased infectivity and evasion of the immune system and highlights suitable or unsuitable antigenic regions for next-generation vaccines and antibody targeting.

## 2. Results

### 2.1. Mutational Landscape of XBB.1.5 S Protein

**Figure 1:**
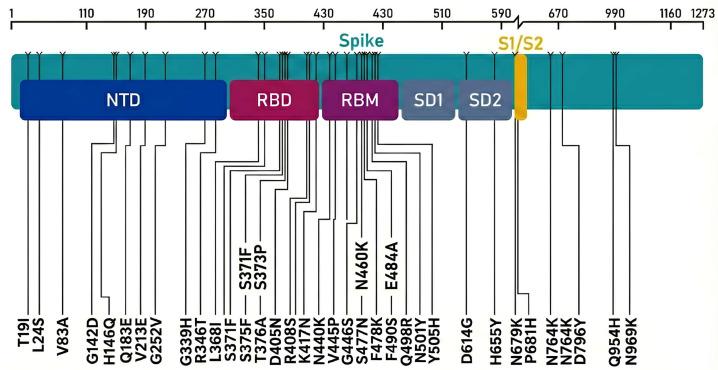
Schematic representation of SARS-CoV-2 S protein XBB.1.5 mutations.

Consensus sequence analysis of the SARS-CoV-2 XBB.1.5 S protein relative to the Wuhan-Hu-1 reference (NC_045512.2; YP_009724390.1) identified 38 amino acid substitutions distributed across S1 and S2 (T19I, L24S, V83A, G142D, H146Q, Q183E, V213E, G252V, G339H, R346T, L368I, S371F, S373P, S375F, T376A, D405N, R408S, K417N, N440K, V445P, G446S, N460K, S477N, T478K, E484A, F486P, F490S, Q498R, N501Y, Y505H, D614G, H655Y, N679K, P681H, N764K, D796Y, Q954H, N969K) (Figure 1).

Twenty-two of these 38 substitutions were localized within the extended RBD, including many positions directly involved in ACE2 binding or targeted by potent class 1–3 neutralizing antibodies (G339H, R346T, S371F, S373P, S375F, T376A, N440K, V445P, G446S, N460K, S477N, T478K, E484A, F486P, F490S, Q498R, N501Y, and Y505H). Several additional mutations cluster in the NTD supersite and S1/S2 region (T19I, L24S, V83A, G142D, H146Q, G183E, V213E, G252V, D614G, H655Y, N679K, P681H), while S2-subunit mutations (N764K, D796Y, Q954H, N969K) localize near the heptad repeat and fusion machinery.

This dense mutational constellation places XBB.1.5 among the most heavily mutated S protein variants described to date, particularly within the RBD, and overlaps with many residues individually associated with increased ACE2 affinity and/or antibody escape in previous Omicron sublineages [9].

### 2.2. Homology Modeling and Overall Structural Impact

Wild-type (Wuhan-Hu-1) and XBB.1.5 S protein ectodomain trimers were modeled using both SWISS-MODEL and RoseTTAFold, followed by energy minimization and quality evaluation using the QMEAN scoring function. The best-scoring models for each variant displayed acceptable global QMEAN Z-scores and residue-level error profiles compatible with comparative modeling in regions with sufficient sequence conservation (QMEAN ≥ 0.7).

**Figure 2:**
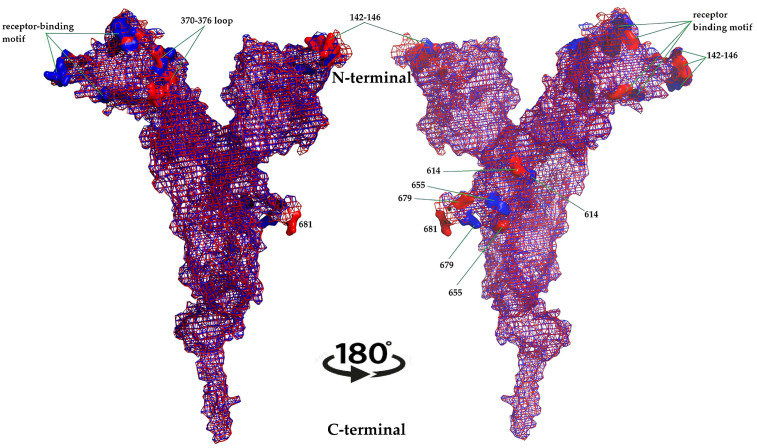
Mesh and surface hybrid illustration of the changes in S protein topology caused by XBB.1.5 mutations. Superimposition representation of the wild-type and XBB.1.5 variant of the S protein. Blue represents the wild-type S protein, and red represents the XBB.1.5 variant of the S protein.

At the global level, the XBB.1.5 S protein models preserved the canonical pre-fusion trimer architecture, with three S1 heads atop S2 stalks. However, mapping the 38 mutations onto the three-dimensional structure revealed dense clustering along the RBD receptor-binding ridge, NTD antigenic supersite, and S1/S2 and S2′ cleavage-proximal regions [16]. The superimposed representation makes the changes in S protein topology caused by mutations visible. Changes in protein topology were particularly noticeable between residues 246–251, 436–441, and 675–683 (Figure 2).

Local structural rearrangements were observed around;

- the 370–376 loop (S371F, S373P, S375F, T376A), which modulates RBD “up/down” transitions and is implicated in altering the conformational ensemble in Omicron sublineages;
- the receptor-binding motif core (G446S, N460K, S477N, T478K, E484A, F486P, Q498R, N501Y, Y505H), which directly contacts ACE2 and is a major target of class 1–3 neutralizing antibodies;
- the NTD supersite around positions 142–146, important for neutralizing NTD-directed antibodies;
- the D614G–H655Y–N679K–P681H region that influences S1/S2 cleavage, fusogenicity, and S protein stability.

The structural context of F486P and Q493 (revertant from R493) in the modeled XBB.1.5 RBD is consistent with experimental structural studies showing that XBB.1.5 maintains or slightly enhances ACE2 binding relative to XBB.1, with P486 contributing to compensatory stabilization of the ACE2–RBD interface despite the loss of hydrophobic interactions associated with F486 in earlier variants.

### 2.3. Predicted B-Cell Epitopes and Antigenic Determinants

B-cell epitope prediction was performed using BepiPred-2.0 for linear epitopes, Emini surface accessibility for solvent exposure, and the Kolaskar–Tongaonkar method for antigenicity, applied to the full S protein sequences of wild-type and XBB.1.5. BepiPred-2.0 identified 28 linear B-cell epitopes in the wild-type S protein and 29 epitopes in XBB.1.5, reflecting both the gain and loss of the predicted epitope segments (Table 1). The mean linear epitope score was calculated as 0.470 (0.183–0.695). Mean BepiPred scores were comparable between variants, but several epitopes shifted in position, length, or score, particularly within the NTD, RBD, and S1/S2 regions where most mutations reside.

Surface accessibility analysis indicated that multiple mutations either increased or decreased the predicted solvent exposure of epitope segments (Table 2). The surface accessibility epitope score was calculated as 1.000 (0.042–6.051). Surface accessibility analysis revealed 30 different epitopes for the wild-type, whereas this value was calculated as 29 for the mutant type. For example, substitutions in the NTD loop region (T19I, L24S, V83A, G142D, H146Q, G183E, V213E, G252V) reshaped contiguous predicted epitopes and altered local accessibility scores, consistent with experimental observations that Omicron NTD mutations reconfigure the NTD supersite and abrogate binding of NTD-specific neutralizing antibodies.

Kolaskar–Tongaonkar antigenicity profiling showed that XBB.1.5 mutations altered the antigenic score distribution along the S protein sequence, with both increases and decreases in predicted antigenicity in key regions (Table 3). The Kolaskar and Tongaonkar antigenicity score was calculated as 1.041 (0.866–1.261). The analysis revealed 46 different epitopes for the wild-type, whereas this value was calculated as 45 for the mutant type. Notably, four antigenic determinants that were highly conserved and antigenic across many pre-Omicron and early Omicron variants showed marked changes in XBB.1.5, suggesting potential disruption of broadly conserved B-cell epitopes.

Using three independent linear B-cell epitope predictors (BepiPred-2.0, Emini surface accessibility, and Kolaskar–Tongaonkar antigenicity), we quantified the differences between wild-type and XBB.1.5 spikes in terms of epitope counts, length distributions, residue-level coverage, turnover, and cross-tool concordance. Overall, epitope counts were highly comparable between the variants across all tools. BepiPred-2.0 predicted 28 wild-type and 29 XBB.1.5 epitopes, Emini predicted 30 wild-type and 29 XBB.1.5 epitopes, and Kolaskar–Tongaonkar predicted 46 wild-type and 45 XBB.1.5 epitopes.

Epitope-length summaries also indicated broadly similar distributions between wild-type and XBB.1.5. For BepiPred-2.0, the mean length was 18.0 aa in wild-type and 16.6 aa in XBB.1.5 (medians 16 vs. 13; ranges 5–62 vs. 5–47). For Emini, the mean length was 9.0 aa in both variants (median 8; range 6–24). For Kolaskar–Tongaonkar, mean length was 9.33 aa (wild-type) versus 9.69 aa (XBB.1.5) (median 8; range 6–36). Consistent with these descriptive statistics, nonparametric comparisons of epitope-length distributions (two-sided Mann–Whitney U test) did not detect significant differences between wild-type and XBB.1.5 for any tool (BepiPred *p* = 0.57, Emini *p* = 0.96, Kolaskar *p* = 0.67), indicating that the mutation set in XBB.1.5 does not substantially shift the overall length profile of predicted linear epitopes. Although epitope counts and lengths were similar, residue-level “coverage” (unique residues falling within any predicted epitope interval) showed modest net changes but notable re-distribution between variants. For BepiPred-2.0, wild-type covered 504 unique residues versus 481 in XBB.1.5 (net: −23). For Emini, wild-type covered 270 residues versus 261 in XBB.1.5 (net −9). For Kolaskar–Tongaonkar, coverage slightly increased from 429 (wild-type) to 436 (XBB.1.5) (net +7).

Importantly, despite these small net differences, residue-level turnover was substantial, indicating marked epitope remodeling between wild-type and XBB.1.5. In BepiPred-2.0, XBB.1.5 gained 104 epitope residues not present in wild-type and lost 127 wild-type specific epitope residues (Jaccard similarity 0.62), with 74.8% of wild-type epitope residues retained in XBB.1.5. In Emini, 109 residues were gained and 118 were lost (Jaccard 0.40), corresponding to 56.3% retention. In Kolaskar–Tongaonkar, 166 residues were gained and 159 were lost (Jaccard 0.45), with 62.9% retention. Collectively, these results show that while the overall number of predicted linear epitope segments remains stable, the locations of the predicted epitope residues can change considerably between wild-type and XBB.1.5.

Next, we assessed internal consistency across tools by comparing the residue-level epitope sets within each variant. The pairwise overlaps were modest, reflecting the different underlying assumptions of propensity-based, accessibility-based, and antigenicity-based predictors. In wild-type, Jaccard overlaps were 0.319 (BepiPred∩Emini), 0.143 (BepiPred∩Kolaskar), and 0.037 (Emini∩Kolaskar). For XBB.1.5, the corresponding overlaps were 0.286, 0.151, and 0.033, respectively.

A “consensus” definition (residues supported by ≥2 tools) yielded 299 consensus residues for wild-type and 289 for XBB.1.5, whereas the strict three-tool intersection was small (fifteen residues in wild-type and nine residues in XBB.1.5). These findings indicate that most predicted epitope residues are tool-specific, whereas a smaller subset is recurrent across methods and may represent more robust candidates for downstream structural mapping and experimental prioritization.

### 2.4. Protein Stability and Dynamics

The impact of each XBB.1.5 mutation on S protein stability was evaluated using four complementary predictors: mCSM, DDMut, DUET, and DynaMut2, which estimate the change in folding free energy ΔΔG between wild-type and mutant (Table 4). The combined evaluation of ΔΔG values obtained from four independent estimating tools (mCSM, DDMut, DUET, and DynaMut2) revealed that the vast majority of mutations showed an average destabilizing trend. Of the 38 mutations, 29 had a negative average ΔΔG and 9 had an positive average ΔΔG; error bars (SD) indicated high inter-tool variability, particularly for some mutations (Supplementary S1). After a two-way one-sample *t*-test per mutation (H0: mean ΔΔG = 0) and Benjamini–Hochberg FDR correction, consistent and statistically significant inter-tool deviations were observed for eight mutations (q < 0.05). Six of these were associated with consistent destabilization: L24S (≈−2.10), V83A (≈−1.09), V213E (≈−1.51), S371F (≈−0.93), T376A (≈−0.50), and F490S (≈−1.89). In contrast, H655Y (≈+1.33) and D796Y (≈+0.45) mutations showed consistent signals of stabilization. While the largest negative mean ΔΔG values were evident for some mutations (L24S and G142D), the wider SD in this group indicated a lower consensus among the instruments. These results were interpreted considering that statistical significance reflects the consistency between predictive instruments, not experimental replicates.

When the functional effects of 38 mutations in the S protein were compared using three different bioinformatics tools (PROVEAN, SIFT, and PolyPhen-2), a significant difference was observed in the tendency to call them “damaging” (Table 5). PROVEAN classified only 2 out of 38 (5.3%) mutations as “deleterious (D)”, while SIFT predicted 4 out of 38 (10.5%) mutations as “affect of function (AF).” PolyPhen-2, on the other hand, evaluated 17 out of 36 (47.2%) mutations as “damaging (D)” (excluding two rows with n/c).

This difference between the three tools was statistically significant using Cochran’s Q test (Q = 26.38, *p* = 1.87 × 10^−6^) and was supported by McNemar tests, particularly showing that PolyPhen-2 called them significantly more often than PROVEAN and SIFT. However, the consensus between the tools was limited. The number of mutations that at least two vehicles identified as “damaging” was 4/36, and this common signal was observed for V83A, Y505H, N764K, and N969K mutations. These results suggest that functional effect predictions can be vehicle-dependent and that the strongest interpretations should be made for mutations supported by a multi-vehicle consensus.

The virus binds to host cell receptors via the S1 subunit of the S proteins and initiates the membrane fusion process necessary for virion translocation to the host cell through S2 subunit activity [17,18]. Two conformational states are observed in the S protein receptor binding region. One is the down formation, where the receptor-binding region is hidden/masked, and the other is the up formation, where the receptor-binding region is accessible and exhibits a less stable structure [19]. In this study, six mutations (N460K, S477N, T478K, E484A, F486P, F490S) were observed in the region between residues 460 and 490, where this event occurs, which the virus uses to evade the body’s defense system, resulting in increased protein instability and mobility (Table 4). Changes in protein topology and atomic interactions in the mutation-related region are thought to contribute to the rapid transmission and increased affinity of the virus [20,21]. Functional impact predictions using PROVEAN, SIFT, and PolyPhen 2 indicated that approximately 44.7% of the analyzed mutations were likely to have deleterious or damaging effects on protein function, particularly those located at or near the ACE2 interface or within dominant neutralizing antibody epitopes. While “deleterious” in this context typically refers to perturbations of the ancestral S protein function, such perturbations may confer a fitness advantage by increasing ACE2 affinity, altering conformational dynamics, or enhancing immune escape.

**Figure 3:**
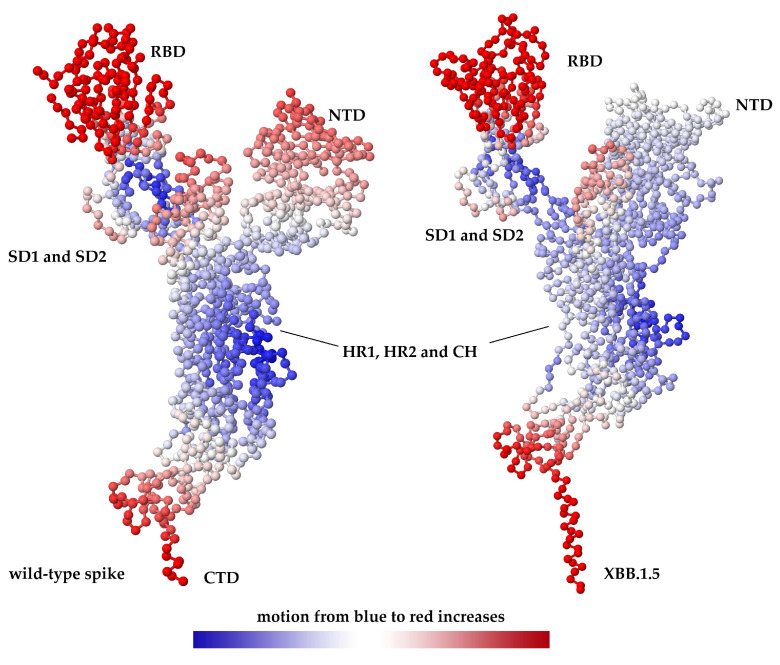
Demonstration of molecular motion of SARS-CoV-2 S protein using a coarse-grained dynamic simulation approach. There is a change in the molecular motion of the S protein following the mutations. In particular, the molecular motion is significantly reduced in NTDs. However, a change in movement was observed at the S1/S2 cleavage site. RBD: recepetor binding domain, NTD: N-terminal domain, CTD: C-terminal domain, SD1: Sub-domain1, SD2: Sub-domain2, HR1: heptad repeat-1, HR2: heptad repeat-2, CH: central helix.

Coarse-grained dynamics were investigated using elastic network models implemented in DynOmics. The cutoff value for the network model nodes was set at 7.3 Å. The correlation between the observed and predicted fluctuations was 0.57 Å^2^ for the wild-type and 0.41 Å^2^ for the variant. These analyses revealed that many XBB.1.5 mutations occur in dynamically active regions that contribute to the collective motions associated with RBD up/down transitions and S1 shedding (Figure 3). In particular, substitutions in the 370–376 loop, RBM core, and S1/S2 cleavage-proximal region were predicted to modulate low-frequency normal modes that control opening of the RBD and exposure of the ACE2-binding surface, consistent with structural and single-particle cryo-EM observations in related Omicron sublineages [22,23].

To move beyond global MD descriptors, we quantified chain-resolved non-covalent interactions at the A–B protomer interface using COCOMAPS 2.0. The overall interaction composition remained broadly similar between the wild-type and XBB.1.5, with proximal contacts remaining the dominant class (47.1% in wild-type vs. 44.8% in XBB.1.5), while small increases were observed for polar vdW contacts (17.6% → 18.8%), CH–O/N contacts (10.2% → 11.3%), hydrogen bonds (5.4% → 5.8%), and salt bridges (1.3% → 1.5%). In agreement with these distributions, the exported contact lists showed only minor changes in absolute interface contact counts (wild-type → XBB.1.5): proximal contacts 217 → 215, polar vdW 81 → 90, apolar vdW 69 → 71, hydrogen bonds 25 → 28, and salt bridges 6 → 7, consistent with subtle interface remodeling rather than gross disruption of the interface.

At the level of key electrostatic anchors, five major salt bridges were conserved across wild-type and XBB.1.5—ASP745–ARG319, GLU868–ARG646, ARG1019–GLU1017, GLU1031–ARG1039, and GLU1151–LYS1149—with shorter interatomic distances in XBB.1.5 (e.g., ARG1019–GLU1017: 4.18 → 3.68 Å; GLU868–ARG646: 4.38 → 3.96 Å), suggesting preserved and potentially strengthened inter-protomer electrostatic coupling. In addition, a new salt bridge was detected in XBB.1.5 (LYS790–GLU702), whereas one peripheral salt bridge displayed a residue-register shift (ARG44 → ARG45 paired with ASP571), consistent with a minor local rearrangement near the N-terminus of the modeled segment.

Hydrogen-bonding patterns were largely retained, with 21 of 25 wild-type residue-pair H-bonds preserved (≈84% conservation) and several showing shorter distances and improved geometry in XBB.1.5 (SER758–GLN965: 3.11 → 2.85 Å; ASN907–ARG1107: 3.06 → 2.81 Å). XBB.1.5 also introduced new H-bond contacts (ARG983–GLY381, LEU981–LYS386, SER982–GLY545), whereas a small number of wild-type H-bonds were lost (MET740–ARG319, TYR873–LEU699). Together, these data indicate that the XBB.1.5 A–B interface undergoes modest repacking, characterized by a slight reduction in non-specific proximity contacts but a compensatory gain in polar and directional interactions.

### 2.5. Integration with Experimental XBB.1.5 Data

Experimental studies have shown that XBB.1.5 combines strong ACE2 binding with substantial escape from neutralizing antibody activity. Surface plasmon resonance and biolayer interferometry measurements indicated that the XBB.1.5 RBD binds human ACE2 with nanomolar affinity, similar to or modestly higher than BA.2 and XBB.1, and higher than the ancestral D614G S protein. Structural analyses reveal that the F486P substitution in XBB.1.5 re-optimizes local packing and preserves ACE2 contacts despite the charge-reversing E484A and other nearby mutations, while the revertant Q493 residue restores favorable hydrogen-bonding interactions with ACE2 residues E35 and H34 [8,24].

Neutralization assays with sera from vaccinated and/or previously infected individuals consistently reported markedly reduced neutralizing titers against XBB and XBB.1.5 compared with earlier Omicron sublineages, even after recent BA.5 breakthrough infection or after updated booster vaccination. XBB.1.5 exhibits broad escape from most authorized therapeutic monoclonal antibodies, with only a limited subset (S309-derived antibodies such as sotrovimab) retaining partial activity, in line with the accumulation of mutations at key monoclonal antibody epitopes (G339H, R346T, G446S, F486P, N460K) [25,26].

The in silico findings of this study—dense mutation clustering at the ACE2 interface and antibody epitopes, reshaped B cell- epitope landscape, and altered stability/dynamics in functionally critical regions—are concordant with these experimental observations and provide a mechanistic structural interpretation of how XBB.1.5 achieves its phenotype.

## 3. Discussion

This study integrated sequence analysis, homology modeling, epitope prediction, stability estimation, and coarse-grained dynamics to characterize how the composite mutational pattern of the SARS-CoV-2 XBB.1.5 S protein variant remodels its structural and antigenic properties. The principal findings are as follows:

- XBB.1.5 harbors 38 S protein substitutions relative to Wuhan-Hu-1, 22 of which reside within the RBD, including many residues directly contacting ACE2 or targeted by potent neutralizing antibodies.
- Homology models indicate preservation of the global prefusion S protein architecture but substantial local remodeling of RBD and NTD loops and the S1/S2 region, consistent with experimental structural data for XBB-lineage spikes.
- B-cell epitope predictions reveal gains and losses of linear epitopes, altered surface accessibility, and changes in antigenicity scores, including disruption of previously conserved antigenic determinants, implying a qualitatively reshaped B-cell antigenic landscape.
- Stability and functional impact predictors show a mosaic of stabilizing and destabilizing mutations, with roughly half predicted to perturb the ancestral S protein function, and elastic-network models suggest that multiple substitutions tune collective motions associated with RBD opening and S1 shedding.
- These in silico observations align with experimental evidence that XBB.1.5 maintains a high ACE2 affinity comparable to BA.2, while exhibiting one of the most extreme neutralizing-antibody escape profiles observed to date.

### 3.1. Structural Basis of Enhanced Receptor Binding

Mutational combinations in XBB.1.5 appear to balance the trade-off between immune escape and receptor engagement, which has shaped SARS-CoV-2 evolution. In earlier Omicron sublineages, certain antibody-evading substitutions in the RBM (E484A and K417N) reduced ACE2 affinity and required compensatory mutations (N501Y and Q498R) to restore or enhance the binding. In XBB.1.5, the revertant Q493 residue reinstates favorable hydrogen bonding with ACE2, while F486P, although removing the aromatic F486 side chain, contributes to local conformational stabilization that supports the ACE2–RBD interface [9,27].

The modeling results highlight how clusters of mutations at 370–376 and 445–486 collectively modulate the conformation and dynamics of the receptor-binding ridge, a key determinant of both ACE2 affinity and antibody epitope exposure. Elastic network analyses suggest that mutations in these regions shift low-frequency normal modes that control the RBD up/down equilibrium and the degree of RBD opening, which in turn influences ACE2 accessibility and the probability of productive receptor engagement. Experimental cryo-EM structures of Omicron spikes, including XBB-lineage variants, support the notion that such mutations alter the distribution of RBD conformers and favor states with enhanced ACE2 occupancy [9,28].

### 3.2. Antigenic Remodeling and Immune Escape

The dense accumulation of mutations in the NTD supersite, RBD core, and RBM is expected to remodel B-cell epitope landscapes by altering the local sequence propensities, segment boundaries, and surface exposure. Accordingly, our linear B-cell epitope predictions indicate that XBB.1.5 does not necessarily reduce the overall number of predicted antigenic segments but rather redistributes antigenic residues are likely to occur along the S protein, implying a qualitative reshaping of antibody-accessible footprints.

To benchmark these immunoinformatics predictions against experimentally reported epitope knowledge, we cross-referenced our findings with IEDB-curated SARS-CoV-2 spike linear epitope datasets, as summarized in recent systematic analyses [29]. In an IEDB-based study of spike linear epitopes, XBB.1.5 signature mutations were reported to map onto 1395 B-cell linear epitopes (32.27% of the IEDB spike B-cell linear epitope set), indicating that roughly one-third of experimentally recorded linear antibody targets contain at least one XBB.1.5 change. Because “affected” here denotes positional overlap rather than direct loss of binding or neutralization, this comparison should be interpreted as a contextual cross-check that nevertheless supports substantial antigenic remodeling in XBB.1.5 [29,30].

Across three linear B-cell epitope prediction approaches (BepiPred-2.0, Emini surface accessibility, and Kolaskar–Tongaonkar antigenicity), wild-type and XBB.1.5 exhibited comparable numbers of predicted epitopes and broadly similar length distributions (Mann–Whitney U tests non-significant across tools), suggesting that the global “quantity” of predicted linear antigenic segments is not dramatically altered by the XBB.1.5 mutation set. Notably, however, the residue-level overlap between wild-type and XBB.1.5 epitope coverage was only moderate (Jaccard ≈ 0.40–0.62 across tools), indicating substantial epitope “turnover” (gain/loss of predicted epitope residues) despite similar global counts. This pattern supports a remodeling scenario in which substitutions shift linear epitope propensities and/or accessibility, potentially changing immunodominant segments without requiring a large change in the total number of predicted epitopes.

These findings are also relevant for interpreting immunoinformatics-driven multi-epitope vaccine designs. Although many efforts have prioritized S-derived epitopes to elicit broad immunity, the rapid evolution of the S protein raises concerns about cross-efficacy across emerging variants [31,32]. For example, epitopes reported as highly conserved across multiple lineages (ELLHAPATV, PYRVVVLSFELLHAP, NATRFASVYAWNRKR, and ERDISTEIYQAGNKP) localize to RBD segments that overlap XBB.1.5 mutational hotspots; thus, even when an epitope’s general region remains targeted, its precise sequence content and predicted segment boundaries can be altered (Table 1, Table 2 and Table 3), which may undermine the assumptions of conservation-based protection [33]. Importantly, inter-tool concordance was limited, reflecting algorithm-specific assumptions (sequence propensity, accessibility, and antigenicity). Therefore, residues supported by ≥2 tools may be viewed as more robust candidates, whereas tool-unique calls should be interpreted conservatively and prioritized for structure-based mapping and experimental validation studies. This qualitative reshaping is consistent with the dramatic loss of neutralization observed for XBB.1.5 against sera from individuals vaccinated with ancestral-strain vaccines, infected with earlier omicron variants, or experiencing hybrid immunity [34].

Many therapeutic monoclonal antibodies targeting class 1 (ACE2-overlapping), class 2 (adjacent to the ACE2 site), class 3 (lateral RBD epitopes), and NTD supersite epitopes lose activity against XBB.1.5 because of mutations at G339H, R346T, G446S, E484A, F486P, N460K, and in the NTD loops [35,36,37]. In this context, the in silico identification of altered antigenic determinants, including turnover within regions previously considered conserved, provides a structural rationale for broad antibody escape and highlights the challenge of designing universal S-based vaccines that rely exclusively on B-cell neutralization.

Simultaneously, experimental data indicate that T-cell recognition of XBB.1.5 S protein remains largely preserved despite extensive antibody escape, likely reflecting the broader and more conserved epitope repertoire of CD4+ and CD8+ responses. Although T-cell epitopes were not explicitly modeled in this study, the concentration of mutations in B-cell-dominated regions suggests that many T-cell epitopes outside the highly variable RBM may remain intact, helping to maintain protection against severe disease even as infection-blocking neutralization wanes [8,38].

### 3.3. Functional Implications of Stability and Dynamics Changes

The mixed pattern of stabilizing and destabilizing mutations in XBB.1.5 underscores that viral fitness does not necessarily correlate with maximal structural stability of the S protein. Instead, an optimal balance between stability and flexibility may favor efficient conformational transitions required for receptor binding, fusion activation, and immune evasion [13,39,40].

Early work on spike S2 evolution provides a useful framework for interpreting XBB.1.5 results beyond antibody escape. Oliva et al. highlighted that mutations can arise in the HR1 “fusion core” a key element of the S2 fusion machinery that refolds and associates with HR2 to form the post-fusion six-helix bundle (6-HB), a conserved architecture essential for membrane fusion and infectivity. In that analysis, the frequent HR1 substitution D936Y was predicted to be structurally consequential because D936 participates in an inter-monomer salt bridge in the post-fusion assembly, motivating comparative dynamics analyses to assess how HR1 mutations may weaken stabilizing interactions in the 6-HB and alter fusion-core behavior [6].

In our XBB.1.5 models, while antigenic remodeling and immune escape are dominated by S1 (NTD/RBD), mutation mapping also indicates changes in the S1/S2 and cleavage-proximal axis, extending into S2-annotated regions (including heptad repeats). Notably, the multi-tool functional effect consensus in our study flagged S2-localized substitutions (N764K and N969K) among the strongest shared “damaging” signals, underscoring that S2 changes may contribute to the phenotype even when most attention is on the RBM. Consistent with this broader view, our elastic network analyses suggest that XBB.1.5 mutations reshape collective motions that couple RBD opening and S1 shedding with S1/S2 dynamics, supporting a model in which variant fitness may reflect coordinated tuning of receptor engagement, immune evasion, and downstream fusion activation rather than S1 changes alone.

Destabilizing mutations in S1 and at the S1/S2 interface can promote S1 shedding and the exposure of the S2 fusion machinery, potentially enhancing fusogenicity; however excessive destabilization risks premature activation and loss of infectivity [41]. Conversely, stabilizing substitutions in the RBD and S2 can preserve the overall integrity of the prefusion trimer while fine-tuning local flexibility [42,43]. Elastic network modeling suggests that XBB.1.5 mutations reshape the collective motions that couple RBD opening with S1/S2 dynamics, potentially optimizing the energetic landscape for ACE2 engagement while minimizing the exposure of vulnerable epitopes.

Global post-MD descriptors (e.g., rmsd/Rg) provide an overall view of structural stability but do not localize how mutations redistribute inter-protomer coupling. By applying COCOMAPS 2.0 to the A–B interface, we showed that XBB.1.5 preserves the core electrostatic “anchor” network connecting neighboring protomers (multiple conserved salt bridges with shorter distances), while simultaneously exhibiting a small shift from bulk proximity packing toward more polar and directional interactions (increased polar vdW/CH–O/N contacts and a slightly larger H-bond and salt-bridge inventory). This pattern is consistent with interface repacking rather than global destabilization: the trimeric architecture remains supported by conserved S2/S1 cross-protomer contacts, yet local rearrangements can tune the mechanical coupling between protomers, potentially influencing conformational transitions linked to receptor engagement, S1 shedding propensity, or exposure of antigenic surfaces.

Notably, the appearance of variant-specific contacts and the gain/loss of selected hydrogen bonds suggest that even modest mutation-driven rearrangements can rewire the local interaction geometry without large changes in the total contact abundance. Such “fine-tuning” is mechanistically plausible for Omicron-descended lineages, where immune escape is often achieved by reshaping accessible surfaces while maintaining the fusion competence. Importantly, COCOMAPS-derived contacts are geometric and threshold-dependent and reflect the analyzed representative structures; therefore, contact differences should be interpreted as structural rationales for altered protomer coupling and prioritized for time-resolved MD contact persistence analysis and experimental validation.

### 3.4. Implications for Vaccine and Antibody Design

The structural and epitope analyses presented here have several implications for the development of next-generation vaccines and therapeutic antibodies:

- Reliance on ancestral strain S protein immunogens is increasingly inadequate in the face of variants such as XBB.1.5, which profoundly remodel key B-cell epitopes. Updated vaccines incorporating XBB-lineage or other highly evolved Omicron spikes are likely necessary to restore robust neutralization breadth.
- Conserved antigenic determinants that remain stable across diverse Omicron sublineages, including XBB.1.5, are attractive targets for broadly neutralizing antibodies and vaccine design. In silico epitope mapping can help identify such regions by excluding highly variable and structurally plastic sites.
- Vaccine strategies that emphasize T-cell epitopes, particularly in more conserved regions of S2 or non-spike proteins, may provide durable protection against severe disease even as S protein-focused neutralization is eroded by antigenic drift.
- Structural insights into how specific mutations, such as F486P and Q493, modulate ACE2 binding and antibody recognition can guide engineering of stabilized immunogens and design of antibody cocktails that target complementary, less mutable epitopes.

### 3.5. Limitations

This study was purely in silico and therefore generated mechanistic hypotheses that require experimental confirmation. First, we did not perform wet-lab validation (e.g., antibody binding assays such as ELISA or SPR/BLI, or authentic/pseudovirus neutralization); therefore, the predicted impacts of XBB.1.5 mutations on antigenicity and antibody escape should be interpreted as putative. Second, our dynamics analysis relies on coarse-grained elastic-network normal-mode models (GNM/ANM) implemented in DynOmics, which are well-suited to capture low-frequency collective motions but do not explicitly represent atomistic side-chain interactions, glycosylation, membrane environment, or solvent, and may therefore miss local energetic effects and transient contacts. Third, the conclusions were derived from the XBB.1.5 consensus sequence and the specific structural templates and parameters used. Because SARS-CoV-2 antigenic evolution is ongoing and epistatic interactions can shift mutational effects across variant backgrounds, generalization to other (especially newly emerging) variants should be made with caution and revisited as new sequences and structures are discovered. Finally, epitope mapping and stability/function predictions depend on the selected algorithms and their training datasets; linear B-cell epitope predictors have limited accuracy, and stability predictors can show inter-method variability. Future studies integrating glycosylated all-atom simulations, structure-based epitope prediction, and targeted experimental assays will be important for validating and refining these findings.

## 4. Materials and Methods

### 4.1. Sequence Retrieval and Variant Definition

The reference S protein sequence for SARS-CoV-2 Wuhan-Hu-1 was obtained from the NCBI database (genomic accession NC_045512.2; S protein accession YP_009724390.1). Consensus S protein sequences representing the XBB.1.5 lineage were retrieved from the NCBI Virus database and aligned to the reference to define a set of characteristic amino acid substitutions [44]. Only high-quality sequences without frameshifts or premature stop codons in the S protein coding region were included. The protein sequence data used in the bioinformatic analyses of wild-type and mutant S proteins are presented in Supplementary S2.

### 4.2. Multiple Sequence Alignment and Domain Mapping

Wild-type and XBB.1.5 S protein sequences were aligned with MAFFT (version 7) using an appropriate scoring matrix (BLOSUM80 and 1PAM) and gap opening penalties (=2), and visualized with Mega11 [45,46]. Mutations were annotated according to standard S protein domain boundaries: signal peptide, NTD, RBD (including RBM), subdomains 1 and 2 (SD1/SD2), S1/S2 cleavage site, S2 fusion peptide, heptad repeats, central helix, connector domain, transmembrane segment, and cytoplasmic tail.

### 4.3. Homology and Deep-Learning-Based Modeling

Homology models for wild-type and variant S proteins were generated using SWISS-MODEL [47]. Templates satisfying threshold values of sequence identity > 97%, sequence similarity > 85, and coverage > 85 were searched using BLAST+ [48] and HHblits [49] for evolutionarily related constructs matching the target sequence in the SWISS-MODEL template library. In protein modeling studies, 6VXX was chosen as the template [50]. Models are built based on the target-template alignment using ProMod3.3.0 [51]. Coordinates that are conserved between the target and template are copied from the template to the model. A fragment library was used to remodel the insertions and deletions, followed by the reconstruction of the side chains. The geometry of the final model was regularized using a force field. The annotation of the template quaternary structure was employed to model the target sequence in its oligomeric state. This approach utilizes a supervised machine learning algorithm, specifically Support Vector Machines (SVM), which integrates interface conservation, structural clustering, and other template characteristics to estimate quaternary structure quality (QSQE) [52]. In parallel, single-stranded SARS-CoV-2 S protein sequences (wild-type and XBB.1.5) were modeled using the RoseTTAFold option of the Robetta structure prediction server [53]. Default settings were used, including automated multiple sequence alignment (MSA) generation and the standard refinement pipeline of the server. Five models were generated for each target (some models were template-dependent), and the final model was selected based on the global confidence/quality outputs (>0.7) reported by the server. To enable direct comparison, the same modeling protocol and settings were applied to the wild-type and XBB.1.5 sequences.

For each variant, multiple models were generated and evaluated, and the best-scoring models based on global quality scores and stereochemical parameters were selected for further analysis.

### 4.4. Model Quality Assessment

The structural quality of the resulting models was assessed using the QMEAN scoring function, which combines residue-level potentials for torsion angles, solvation, and long-range interactions into a normalized Z-score that reflects model agreement with high-resolution experimental structures of similar size [54]. Local QMEAN scores were inspected to identify poorly modeled regions that might limit the reliability of detailed structural interpretations.

### 4.5. B-Cell Epitope and Antigenicity Prediction

Linear B-cell epitopes were predicted using BepiPred-2.0, which applies a random-forest classifier trained on experimentally determined antibody–antigen complexes to sequence-derived features [55]. Default thresholds (=0.5) were used to define epitope residues. Linear epitopes that met the threshold of ≥5 amino acid residues were considered.

Surface accessibility was evaluated using the Emini method, which estimates the probability of each residue being solvent-exposed based on normalized surface accessibility scales derived from known protein structures [56]. Residues with high surface accessibility scores were considered more likely to be part of antibody-accessible epitopes.

Antigenicity profiles were calculated using the Kolaskar–Tongaonkar semi-empirical method, which integrates physicochemical properties and residue frequencies in experimentally defined antigenic regions to predict the antigenic determinants [57]. Regions with antigenicity scores above the method-specific threshold (=0.5) were considered putative antigenic sites.

Linear B-cell epitopes were predicted independently for the wild-type and XBB.1.5 S protein sequences using three tools (BepiPred-2.0, Emini surface accessibility, and Kolaskar–Tongaonkar antigenicity). For each tool and variant, we summarized (i) the number of predicted epitope intervals and (ii) epitope length (amino acids), reporting the mean, median, and range. Differences in epitope length distributions between variants were tested using the two-sided Mann–Whitney U test (α = 0.05). To quantify epitope “coverage”, each epitope interval was expanded to residue-level sets (all positions between start and end), merged to remove overlaps, and the number of unique covered residues was computed per tool/variant. Epitope remodeling (“turnover”) between wild-type and XBB.1.5 was assessed at the residue level by calculating gained residues (XBB-only), lost residues (wild-type-only), and similarity using the Jaccard index J=∣A∩B∣/∣A∪B∣, where A and B denote the residue sets covered by epitopes in wild-type and XBB.1.5, respectively; retention was additionally reported as ∣A∩B∣/∣A∣. Finally, within each variant, inter-tool concordance was quantified by pairwise Jaccard indices between residue-level sets and by counting the “consensus” residues supported by ≥2 tools (and the three-tool intersection).

To contextualize immunoinformatics-based epitope predictions with experimental evidence, we consulted IEDB-curated SARS-CoV-2 spike linear epitope resources and recent IEDB-wide analyses that quantify the fraction of curated linear epitopes intersected by XBB.1.5-characterizing mutations [29,30].

### 4.6. Stability and Functional Impact Prediction

The impact of individual amino acid substitutions on S protein stability was assessed using:

- mCSM, which encodes the wild-type structural environment of a residue as graph-based signatures and uses machine learning to predict ΔΔG [58],
- DDMut, a deep-learning model trained on a large dataset of experimentally measured stability changes [59],
- DUET, which combines mCSM and the SDM (site-directed mutator) potential to provide consensus ΔΔG estimates [60],
- DynaMut2, which integrates normal-mode analysis with machine learning to estimate both stability changes and alterations in protein flexibility upon mutation [61].

To quantitatively compare the effect of mutations on protein stability, ΔΔG (kcal·mol^−1^) values obtained from four independent predictor tools (mCSM, DDMut, DUET, and DynaMut2) were evaluated for each mutation. The mean ΔΔG between tools was calculated for each mutation, and the standard deviation (SD) and standard error (SEM = SD/√*n*, *n* = 4) were derived to show uncertainty and inter-method variability. To test whether the stability change deviated significantly from zero, a two-way one-sample *t*-test was applied for each mutation to test the hypothesis H0: mean ΔΔG = 0, and the corresponding *p*-values were reported. To control for false positives from multiple comparisons, *p*-values were corrected using the Benjamini–Hochberg false discovery rate (FDR) method, and q-values were calculated for all tests. In the graphs, error bars are shown as SD (where appropriate) to represent the variability between predictor tools; negative mean ΔΔG values are interpreted as destabilization, while positive values are interpreted as stabilization.

To quantitatively compare the potential effects of mutations on protein function, the outputs of three different bioinformatics tools (PROVEAN, SIFT, and PolyPhen-2) were evaluated together for each mutation [62,63,64]. First, the scores and classes obtained from each instrument were standardized and converted into binary results. For PROVEAN, the “deleterious (D)” category and for SIFT, the “affect of function (AF)” category were coded as “damaging,” while other classes were coded as “non-damaging.” For PolyPhen-2, the “damaging (D)” result was considered “damaging.” Rows with “n/c” were treated as missing data in relevant analyses. The rates of “damaging” calls made by the instruments were calculated as percentages based on valid observations, and 95% confidence intervals for these rates were reported using the Wilson method. Whether these binary calls, which are repeated/dependent measurements on the same mutations, differed among the instruments was evaluated using Cochran’s Q test in rows where the three instruments had common data. Systematic differences between pairs of instruments were tested using the McNemar exact test. Inter-instrument agreement was summarized using the raw agreement rate and Cohen’s kappa coefficient for binary classes. To ensure a directional comparison of continuous scores, PROVEAN scores were converted to a “severity” indicator (-score), SIFT scores were inverted to 1-score, and PolyPhen-2 scores were used directly. Inter-instrumental ranking consistency was examined using Spearman’s correlation. Finally, to evaluate consensus based on mutations, mutations that were identified as “damaging” by at least two instruments were defined as “consensus-damaging”.

### 4.7. Coarse-Grained Dynamics: Elastic-Network Models

To examine how clusters of mutations affect collective protein motions, coarse-grained dynamics simulations were conducted using DynOmics, which implements the Gaussian Network Model (GNM) and Anisotropic Network Model (ANM) elastic network approaches [65]. In these models, Cα atoms are connected by springs within a cutoff distance, and normal-mode analysis is used to compute low-frequency modes that describe large-scale motions around the equilibrium structure. In the context of ANM, the threshold for internode interactions was established at 15.0 Å, with a γ value of 1.0. Conversely, for the GNM, a threshold of 10.0 Å and a γ value of 1.0 were employed.

The slowest modes were analyzed for mean-square fluctuations (MSFs), computed as ${\mathrm{MSF}}_{i}=\frac{k_{B}T}{\gamma }\sum_{k=1}^{m}(L_{ik}{)}^{2}/\lambda_{k}$, where $L_{ik}$ denotes the k-th eigenvector component for residue *i*, $\lambda_{k}$ the eigenvalue, m the mode cutoff, $k_{B}T=0.6$ kcal·mol^−1^ at RT, and individual residue collectivity quantified via $c_{i}=|\sum_{j=1}^{N}L_{ij}|$. Vibrational entropy contributions per mode were evaluated as $S_{k}=-\frac{1}{2}k_{B}\ln(2\pi \lambda_{k})+\mathrm{const}$, summed across modes for total configurational entropy.

For the mutants, identical parameters ensured direct comparability, with ΔMSF profiles highlighting mutation-induced dynamic perturbations in functional regions, such as binding interfaces. All computations used the default server settings without membrane or environmental adjustments, processing PDB structures post-hydrogen addition, and energy minimization.

### 4.8. Chain-Specific İnter-Protomer İnteraction Profiling

Chain-specific intermolecular contacts between spike protomers were characterized using the COCOMAPS 2.0 web server [66] by analyzing the A–B chain interface for both wild-type and XBB.1.5 models. Interacting residues were selected using a 5.0 Å cut-off. The contact definitions and thresholds were set as follows: H-bond distance 3.9 Å with minimum DHA angle 90°; salt-bridge distance 4.5 Å; water-mediated distance 3.9 Å; disulfide distance 3.6 Å; π–π distance 5.5 Å (θ = 80°, γ = 90°); cation–π, anion–π, lone pair–π, and amino–π distances 5.0 Å; CH–O/N distance 3.6 Å with CCH–O/N angle 110°; CH–π distance 4.5 Å (θ1 = 120°, θ2 = 30°); N/O/SH–π distance 4.5 Å (θ1 = 120°, θ2 = 30°); apolar and polar tolerance values 0.5.

## 5. Conclusions

The SARS-CoV-2 Omicron XBB.1.5 S protein illustrates how extensive sequence diversification can be leveraged to preserve receptor engagement while reshaping antigenicity. Our integrated computational workflow indicates that the prefusion trimeric architecture is globally maintained, yet mutations are concentrated in the NTD and RBD and drive local structural rearrangements in regions that govern ACE2 binding, antibody recognition and S1/S2 activation.

Across three independent linear B-cell epitope predictors, XBB.1.5 retained broadly similar epitope counts and length distributions compared with the wild-type but showed only moderate residue-level overlap, consistent with substantial epitope “turnover” rather than a simple net loss of antigenic segments. This remodeling is further supported by an IEDB-based cross-check indicating that a sizable fraction of curated spike linear B-cell epitopes overlap with XBB.1.5 signature mutation sites, underscoring the likelihood that antibody footprints are redistributed across the spike surface. Together, these results support a model in which immune evasion can arise through qualitative re-patterning of accessible and antigenic residues, even when the global epitope totals remain comparable.

Beyond global stability descriptors, chain-specific interface analysis with COCOMAPS suggests that XBB.1.5 preserves the core A–B inter-protomer electrostatic “anchor” network while exhibiting modest repacking, including slight enrichment of polar/directional contacts and variant-specific gains/losses of selected hydrogen bonds and/or salt bridges. This pattern argues against gross destabilization of the trimer and instead supports fine-tuning of inter-protomer coupling that may influence conformational transitions linked to receptor engagement and S1 shedding. Overall, our findings align with the reported experimental observations of strong ACE2 binding coupled with profound neutralizing-antibody escape in XBB-lineage variants and reinforce the need for updated vaccine antigens and therapeutic antibodies explicitly considering the structural and antigenic landscape of highly evolved Omicron sublineages. The proposed framework can be readily applied to newly emerging variants to rapidly generate mechanistic hypotheses and prioritize conserved targets for downstream structure-guided design and experimental validation.

## Figures and Tables

**Table 1:** Linear B-cell epitopes for wild-type and XBB.1.5.

	Wild Type				XBB.1.5			
	Initial	Final	Length	Peptide	Initial	Final	Length	Peptide
1	13	37	25	SQCVNLTTRTQLPPAYTNSFTRGVY	13	27	15	SQCVNLITRTQSYTN
2	59	81	23	FSNVTWFHAIHVSGTNGTKRFDN	65	83	19	IHVSGTNGTKRFDNPALPF
3	138	154	17	DPFLGVYYHKNNKSWME	134	151	18	NDPFLDVYQKNNKSWMES
4	177	189	13	MDLEGKQGNFKNL	172	184	13	LMDLEGKEGNFKN
5	206	221	16	KHTPINLVRDLPQGFS	204	216	13	TPINLERDLPQGF
6	250	260	11	TPGDSSSGWTA	244	256	13	YLTPVDSSSGWTA
7	304	322	19	KSFTVEKGIYQTSNFRVQP	300	307	8	KSFTVEKG
8	329	363	35	FPNITNLCPFGEVFNATRFASVYA WNRKRISNCVA	309	318	10	YQTSNFRVQP
9	369	393	25	YNSASFSTFKCYGVSPTKLNDLCFT	326	361	36	PNITNLCPFHEVFNATTFASVYA WNRKRISNCVADY
10	404	426	23	GDEVRQIAPGQTGKIADYNYKLP	367	386	20	FAPFFAFKCYGVSPTKLNDL
11	440	501	62	NLDSKVGGNYNYLYRLFRKSNLKPFERDIST EIYQAGSTPCNGVEGFNCYFPLQSYGFQPTN	405	416	12	QIAPGQTGNIAD
12	516	536	21	ELLHAPATVCGPKKSTNLVKN	435	447	13	NKLDSKPSGNYNY
13	555	562	8	SNKKFLPF	453	499	47	RKSKLKPFERDISTEIYQAGNKPC NGVAGPNCYSPLQSYGFRPTYGV
14	602	606	5	TNTSN	511	531	21	FELLHAPATVCGPKKSTNLVK
15	616	632	17	NCTEVPVAIHADQLTPT	551	558	8	SNKKFLPF
16	634	644	11	RVYSTGSNVFQ	598	602	5	TNTSN
17	656	666	11	VNNSYECDIPI	612	628	17	NCTEVPVAIHADQLTPT
18	672	690	19	ASYQTQTNSPRRARSVASQ	634	640	7	TGSNVFQ
19	695	710	16	YTMSLGAENSVAYSNN	650	662	13	EYVNNSYECDIPI
20	773	779	7	EQDKNTQ	669	686	18	SYQTQTKSHRRARSVASQ
21	786	800	15	KQIYKTPPIKDFGGF	691	705	15	YTMSLGAENSVAYSN
22	807	814	8	PDPSKPSK	768	775	8	VEQDKNTQ
23	828	842	15	LADAGFIKQYGDCLG	779	811	33	AQVKQIYKTPPIKYFGGFNF SQILPDPSKPSKR
24	988	992	5	EAEVQ	824	838	15	LADAGFIKQYGDCLG
25	1035	1043	9	GQSKRVDFC	984	988	5	EAEVQ
26	1107	1118	12	RNFYEPQIITTD	1030	1039	10	LGQSKRVDFC
27	1133	1172	40	VNNTVYDPLQPELDSFKEELDKYFKN HTSPDVDLGDISGI	1103	1114	12	RNFYEPQIITTD
28	1252	1267	16	SCCKFDEDDSEPVLKG	1129	1168	40	VNNTVYDPLQPELDSFKEEL DKYFKNHTSPDVDLGDISGI
29					1249	1265	17	CCKFDEDDSEPVLKGVK

**Table 2:** B-cell surface accessibility epitopes for wild-type and XBB.1.5 variant.

	Wild Type				XBB.1.5			
	Initial	Final	Length	Peptide	Initial	Final	Length	Peptide
1	18	32	15	LTTRTQLPPAYTNSF	20	29	10	TRTQSYTNSF
2	35	43	9	GVYYPDKVF	32	40	9	GVYYPDKVF
3	73	80	8	TNGTKRFD	70	77	8	TNGTKRFD
4	110	115	6	LDSKTQ	107	112	6	LDSKTQ
5	144	153	10	YYHKNNKSWM	140	149	10	VYQKNNKSWM
6	179	185	7	LEGKQGN	175	181	7	LEGKEGN
7	202	208	7	KIYSKHT	198	204	7	KIYSKHT
8	250	255	6	TPGDSS	207	212	6	NLERDL
9	278	284	7	KYNENGT	274	279	6	KYNENG
10	314	323	10	QTSNFRVQPT	314	319	6	FRVQPT
11	352	357	6	AWNRKR	415	424	10	ADYNYKLPDD
12	419	428	10	ADYNYKLPDD	433	448	16	NSNKLDSKPSGNYNYL
13	437	442	6	NSNNLD	451	464	14	LFRKSKLKPFERDI
14	455	468	14	LFRKSNLKPFERDI	491	496	6	YGFRPT
15	495	500	6	YGFQPT	565	576	12	IADTTDAVRDPQ
16	569	581	13	IADTTDAVRDPQT	597	602	6	GTNTSN
17	601	606	6	GTNTSN	623	632	10	DQLTPTWRVY
18	627	636	10	DQLTPTWRVY	651	656	6	YVNNSY
19	655	660	6	HVNNSY	670	680	11	YQTQTKSHRRA
20	674	685	12	YQTQTNSPRRAR	769	775	7	EQDKNTQ
21	773	779	7	EQDKNTQ	782	790	9	KQIYKTPPI
22	786	794	9	KQIYKTPPI	804	813	10	DPSKPSKRSF
23	808	817	10	DPSKPSKRSF	911	916	6	VLYENQ
24	914	920	7	NVLYENQ	1064	1072	9	VPAQEKNFT
25	1068	1076	9	VPAQEKNFT	1101	1107	7	TQRNFYE
26	1105	1111	7	TQRNFYE	1135	1158	24	DPLQPELDSFKEELDKY FKNHTSP
27	1139	1162	24	DPLQPELDSFKEEL DKYFKNHTSP	1175	1182	8	IQKEIDRL
28	1179	1186	8	IQKEIDRL	1198	1206	9	ELGKYEQYI
29	1202	1210	9	ELGKYEQYI	1252	1257	6	FDEDDS
30	1256	1261	6	FDEDDS				

**Table 3:** Kolaskar and Tongaonkar B-cell epitopes for wild-type and XBB.1.5.

	Wild Type				XBB.1.5			
	Initial	Final	Length	Peptide	Initial	Final	Length	Peptide
1	4	18	15	FLVLLPLVSSQCVNL	4	18	15	FLVLLPLVSSQCVNL
2	34	41	8	RGVYYPDK	31	38	8	RGVYYPDK
3	44	51	8	RSSVLHST	41	48	8	RSSVLHST
4	53	60	8	DLFLPFFS	50	57	8	DLFLPFFS
5	65	70	6	FHAIHV	62	67	6	FHAIHV
6	81	87	7	NPVLPFN	112	118	7	QSLLIVN
7	115	121	7	QSLLIVN	122	131	10	NVVIKVCEFQ
8	125	134	10	NVVIKVCEFQ	133	141	9	CNDPFLDVY
9	136	146	11	CNDPFLGVYYH	164	170	7	FEYVSQP
10	168	174	7	FEYVSQP	219	226	8	LEPLVDLP
11	210	216	7	INLVRDL	235	248	14	QTLLALHRSYLTPV
12	223	230	8	LEPLVDLP	259	266	8	AAYYVGYL
13	239	248	10	QTLLALHRSY	268	274	7	PRTFLLK
14	263	270	8	AAYYVGYL	284	291	8	AVDCALDP
15	272	278	7	PRTFLLK	329	337	9	TNLCPFHEV
16	288	295	8	AVDCALDP	355	368	14	SNCVADYSVIYNFA
17	333	339	7	TNLCPFG	370	381	12	FFAFKCYGVSPT
18	359	371	13	SNCVADYSVLYNS	426	431	6	TGCVIA
19	376	385	10	TFKCYGVSPT	481	490	10	GPNCYSPLQS
20	430	435	6	TGCVIA	498	523	26	GVGHQPYRVVVLSFELLHAPATVCGP
21	488	495	8	CYFPLQSY	588	595	8	FGGVSVIT
22	505	527	23	YQPYRVVVLSFELLHAPATV CGP	603	611	9	QVAVLYQGV
23	592	599	8	FGGVSVIT	613	623	11	CTEVPVAIHAD
24	607	615	9	QVAVLYQDV	643	649	7	AGCLIGA
25	617	627	11	CTEVPVAIHAD	663	670	8	GAGICASY
26	647	653	7	AGCLIGA	683	689	7	VASQSII
27	667	674	8	GAGICASY	719	726	8	TTEILPVS
28	687	693	7	VASQSII	731	737	7	SVDCTMY
29	723	730	8	TTEILPVS	746	761	16	SNLLLQYGSFCTQLKR
30	735	741	7	SVDCTMY	777	784	8	VFAQVKQI
31	750	763	14	SNLLLQYGSFCTQL	799	804	6	SQILPD
32	781	788	8	VFAQVKQI	833	839	7	YGDCLGD
33	803	808	6	SQILPD	843	849	7	RDLICAQ
34	837	843	7	YGDCLGD	854	860	7	LTVLPPL
35	847	853	7	RDLICAQ	869	876	8	YTSALLAG
36	858	864	7	LTVLPPL	944	950	7	LQDVVNH
37	873	880	8	YTSALLAG	955	962	8	LNTLVKQL
38	959	966	8	LNTLVKQL	969	975	7	ISSVLND
39	973	979	7	ISSVLND	999	1007	9	SLQTYVTQQ
40	1003	1011	9	SLQTYVTQQ	1026	1033	8	SECVLGQS
41	1030	1037	8	SECVLGQS	1053	1066	14	PHGVVFLHVTYVPA
42	1057	1070	14	PHGVVFLHVTYVPA	1075	1081	7	PAICHDG
43	1079	1085	7	PAICHDG	1119	1128	10	SGNCDVVIGI
44	1123	1132	10	SGNCDVVIGI	1170	1175	6	ASVVNI
45	1174	1179	6	ASVVNI	1217	1252	36	IAGLIAIVMVTIMLCCMTSCCSCLKGCCS CGSCCKF
46	1221	1256	36	IAGLIAIVMVTIMLCCMTSCCSCLKGCCSC				

**Table 4:** Effects of XBB.1.5 spike mutations on protein stability, with summary statistics and FDR-adjusted *p* values.

Mutation	mCSM	DDMut	DUET	DynaMut2	Mean_ΔΔG	SD	SEM	*p*_Value	q_FDR_BH	Direction
T19I	−0.185	0.05	0.294	−0.21	−0.013	0.236	0.118	0.9207	0.9207	Destabilizing
L24S	−2.604	−0.89	−2.902	−2.0	−2.099	0.889	0.445	0.01799	0.07597	Destabilizing
V83A	−1.12	−1.34	−1.255	−0.65	−1.091	0.308	0.154	0.00577	0.03132	Destabilizing
G142D	−2.162	−0.26	−2.342	−1.33	−1.523	0.951	0.475	0.04914	0.1245	Destabilizing
H146Q	−0.875	0.3	−0.973	−0.19	−0.434	0.601	0.3	0.2439	0.309	Destabilizing
Q183E	−0.009	0.03	0.455	0.14	0.154	0.21	0.105	0.2393	0.309	Stabilizing
V213E	−1.912	−1.61	−1.542	−0.99	−1.514	0.384	0.192	0.004263	0.027	Destabilizing
G252V	−0.422	−1.53	0.008	−1.11	−0.764	0.688	0.344	0.113	0.2059	Destabilizing
G339H	−0.98	−0.32	−0.599	−1.06	−0.74	0.345	0.172	0.02324	0.08831	Destabilizing
R346T	−0.34	0.0	−0.26	−0.16	−0.19	0.147	0.073	0.08082	0.1807	Destabilizing
L368I	0.233	−0.32	0.485	0.08	0.12	0.337	0.169	0.5296	0.575	Stabilizing
S371F	−1.142	−0.94	−0.77	−0.88	−0.933	0.156	0.078	0.001259	0.027	Destabilizing
S373P	−0.497	0.01	−0.542	−0.15	−0.295	0.268	0.134	0.1154	0.2059	Destabilizing
S375F	−0.985	−0.19	−0.484	−0.69	−0.587	0.335	0.168	0.03939	0.1152	Destabilizing
T376A	−0.669	−0.41	−0.461	−0.45	−0.498	0.116	0.058	0.003365	0.027	Destabilizing
D405N	−1.047	−0.04	−0.817	−0.98	−0.721	0.464	0.232	0.05302	0.1259	Destabilizing
R408S	−0.277	−0.06	−0.241	−0.38	−0.24	0.133	0.067	0.03699	0.1152	Destabilizing
K417N	−0.849	−0.2	−0.58	−0.56	−0.547	0.266	0.133	0.0261	0.09017	Destabilizing
N440K	0.207	0.2	0.544	0.01	0.24	0.222	0.111	0.1192	0.2059	Stabilizing
V445P	−0.302	−0.21	−0.145	0.21	−0.112	0.224	0.112	0.3919	0.4512	Destabilizing
G446S	−0.701	−0.08	−0.516	−0.11	−0.352	0.306	0.153	0.1052	0.2059	Destabilizing
N460K	−0.022	0.02	0.355	−0.2	0.038	0.232	0.116	0.763	0.7836	Stabilizing
S477N	−0.13	0.02	0.309	0.21	0.102	0.196	0.098	0.3731	0.4431	Stabilizing
T478K	−0.511	−0.18	−0.132	0.02	−0.201	0.224	0.112	0.1706	0.2701	Destabilizing
E484A	−0.589	−0.12	−0.412	0.11	−0.253	0.31	0.155	0.2011	0.2978	Destabilizing
F486P	−0.525	−0.02	−0.474	0.05	−0.242	0.299	0.15	0.2038	0.2978	Destabilizing
F490S	−2.19	−1.79	−2.116	−1.47	−1.892	0.33	0.165	0.00143	0.027	Destabilizing
Q498R	−0.274	−1.05	0.102	−0.1	−0.33	0.504	0.252	0.2808	0.3442	Destabilizing
N501Y	−0.392	−0.82	−0.217	−0.3	−0.432	0.268	0.134	0.04846	0.1245	Destabilizing
Y505H	−0.603	−1.01	−0.191	−0.1	−0.476	0.418	0.209	0.1071	0.2059	Destabilizing
D614G	−0.68	−0.26	−0.024	−0.19	−0.289	0.279	0.14	0.1306	0.2158	Destabilizing
H655Y	1.061	1.23	1.196	1.82	1.327	0.337	0.168	0.004262	0.027	Stabilizing
N679K	0.199	−0.09	0.497	0.15	0.189	0.241	0.121	0.2149	0.3024	Stabilizing
P681H	−1.615	−1.03	−0.964	−1.16	−1.192	0.293	0.147	0.003893	0.027	Destabilizing
N764K	−0.233	−0.63	0.301	0.03	−0.133	0.397	0.198	0.5505	0.581	Destabilizing
D796Y	0.234	0.57	0.538	0.46	0.45	0.152	0.076	0.009514	0.04519	Stabilizing
Q954H	−0.58	0.31	−0.322	−0.04	−0.158	0.382	0.191	0.4688	0.524	Destabilizing
N969K	0.243	0.02	0.678	0.01	0.238	0.313	0.156	0.2256	0.3061	Stabilizing

**Table 5:** Functional impact of XBB.1.5 spike mutations, including consensus classification (≥2 tools).

Mutation	PROVEAN		SIFT		PolyPhen		Tools	Damaging Calls	Consensus Damaging (≥2)
	Score	Call	Score	Call	Score	Call			
T19I	−0.7	N	0.1	T	0.014	B	3	0	No
L24S	0.088	N	0.66	T	0.088	B	3	0	No
V83A	−0.644	N	0.02	AF	0.925	D	3	2	Yes
G142D	−0.277	N	0.18	T	0.03	B	3	0	No
H146Q	−0.037	N	0.39	T	0.027	B	3	0	No
Q183E	−0.399	N	0.86	T	0.052	B	3	0	No
V213E	−1.074	N	1	T	0.903	D	3	1	No
G252V	0.119	N	0.17	T	-	n/c	2	0	No
G339H	−0.878	N	0.16	T	0.994	D	3	1	No
R346T	0.303	N	0.34	T	0.957	D	3	1	No
L368I	−0.286	N	0.11	T	0.758	D	3	1	No
S371F	−0.426	N	0.26	T	1	D	3	1	No
S373P	1.211	N	0.38	T	0.993	D	3	1	No
S375F	−0.239	N	0.07	T	1	D	3	1	No
T376A	−0.931	N	0.33	T	0.994	D	3	1	No
D405N	−0.199	N	0.27	T	0.781	D	3	1	No
R408S	0.841	N	0.87	T	1	D	3	1	No
K417N	0.27	N	0.56	T	0.747	D	3	1	No
N440K	−1.663	N	0.7	T	0.003	B	3	0	No
V445P	−0.171	N	0.32	T	0.066	B	3	0	No
G446S	0.514	N	0.79	T	0.001	B	3	0	No
N460K	0.006	N	1	T	0	B	3	0	No
S477N	−0.034	N	0.84	T	0.001	B	3	0	No
T478K	−0.524	N	0.75	T	0	B	3	0	No
E484A	0.732	N	0.51	T	0.72	D	3	1	No
F486P	−0.761	N	0.15	T	0.025	B	3	0	No
F490S	0.401	N	0.17	T	0.42	B	3	0	No
Q498R	0.056	N	0.39	T	0.003	B	3	0	No
N501Y	−0.09	N	0.09	T	0.206	B	3	0	No
Y505H	−0.667	N	0.03	AF	1	D	3	2	Yes
D614G	0.598	N	0.62	T	0.009	B	3	0	No
H655Y	−0.814	N	0.5	T	0.001	B	3	0	No
N679K	0.252	N	0.53	T	0.009	B	3	0	No
P681H	0.06	N	0.03	AF	-	n/c	2	1	No
N764K	−2.836	D	0	AF	0.997	D	3	3	Yes
D796Y	0.044	N	1	T	0.007	B	3	0	No
Q954H	−2.038	N	0.08	T	0.957	D	3	1	No
N969K	−3.066	D	0.09	T	1	D	3	2	Yes

D: deleterious, N: natural, T: tolerable, B: benign, AF: affect of function, n/c: not calculated.

## Data Availability

The raw data supporting the conclusions of this article will be made available by the authors on request.

## Supplementary Materials

The following supporting information can be downloaded at: https://www.mdpi.com/article/10.3390/ijms27041940/s1.

## Abbreviations

The following abbreviations are used in this manuscript:

ACE2	Angiotensin-converting enzyme-2
ANM	Anisotropic Network Model
BLOSUM80	BLOcks SUbstitution Matrix 80
CD4	Cluster of Differentiation 4
CD8	Cluster of Differentiation 8
COVID-19	Coronavirus disease 2019
CTD	C-terminal domain
Cryo-EM	Cryo-electron microscopy
GNM	Gaussian Network Model
HR1	Heptade repeat 1
HR2	Heptade repeat 2
MAFFT	Multiple Alignment using Fast Fourier Transform
mCSM	mutation Cutoff Scanning Matrix
NTD	N-terminal domain
Provean	Protein Variation Effect Analyzer
RBD	Receptor-binding domain
RBM	Receptor-binding motif
RNA	Ribonucleic acid
S1	Spike subunit 1
S2	Spike subunit 2
SD1	Subdomain 1
SD2	Subdomain 1
SARS-CoV-2	Severe acute respiratory syndrome coronavirus-2

## References

[1] Shang J., Wan Y., Luo C., Ye G., Geng Q., Auerbach A., Li F. (2020). Cell Entry Mechanisms of SARS-CoV-2. Proc. Natl. Acad. Sci. USA.

[2] Akbulut E. (2021). Mutations in the SARS CoV-2 Spike Protein May Cause Functional Changes in the Protein Quaternary Structure. Turk. J. Biochem..

[3] COVID—Coronavirus Statistics—Worldometer. https://www.worldometers.info/coronavirus/.

[4] Aliper E.T., Krylov N.A., Nolde D.E., Polyansky A.A., Efremov R.G. (2022). A Uniquely Stable Trimeric Model of SARS-CoV-2 Spike Transmembrane Domain. Int. J. Mol. Sci..

[5] Yajima H., Nomai T., Okumura K., Maenaka K., Ito J., Hashiguchi T., Sato K., Genotype to Phenotype Japan (G2P-Japan) Consortium (2024). Molecular and Structural Insights into SARS-CoV-2 Evolution: From BA. 2 to XBB Subvariants. mBio.

[6] Oliva R., Shaikh A.R., Petta A., Vangone A., Cavallo L. (2021). D936Y and Other Mutations in the Fusion Core of the SARS-CoV-2 Spike Protein Heptad Repeat 1: Frequency, Geographical Distribution, and Structural Effect. Molecules.

[7] Yue C., Song W., Wang L., Jian F., Chen X., Gao F., Shen Z., Wang Y., Wang X., Cao Y. (2023). ACE2 Binding and Antibody Evasion in Enhanced Transmissibility of XBB. 1.5. Lancet Infect. Dis..

[8] Mannar D., Saville J.W., Poloni C., Zhu X., Bezeruk A., Tidey K., Ahmed S., Tuttle K.S., Vahdatihassani F., Cholak S. (2024). Altered Receptor Binding, Antibody Evasion and Retention of T Cell Recognition by the SARS-CoV-2 XBB. 1.5 Spike Protein. Nat. Commun..

[9] Zhang Q.E., Lindenberger J., Parsons R.J., Thakur B., Parks R., Park C.S., Huang X., Sammour S., Janowska K., Spence T.N. (2024). SARS-CoV-2 Omicron XBB Lineage Spike Structures, Conformations, Antigenicity, and Receptor Recognition. Mol. Cell.

[10] Yang J., Hong W., Lei H., He C., Lei W., Zhou Y., Zhao T., Alu A., Ma X., Li J. (2023). Low Levels of Neutralizing Antibodies against XBB Omicron Subvariants after BA. 5 Infection. Signal Transduct. Target. Ther..

[11] Reinholm A., Khan H., Laakso T., Maljanen S., Jalkanen P., Gunell M., Kallonen T., Österlund P., Ritvos O., Nousiainen A. (2025). Corrigendum to “Long-Term Neutralization Capacity of Vaccine and Breakthrough Infection Induced SARS-CoV-2 Specific Antibodies against Omicron Subvariants BA. 2, XBB. 1.5, and JN. 1”. Vaccine.

[12] Springer D.N., Camp J.V., Aberle S.W., Deutsch J., Lammel O., Weseslindtner L., Stiasny K., Aberle J.H. (2024). Neutralization of SARS-CoV-2 Omicron XBB. 1.5 and JN. 1 Variants after COVID-19 Booster-vaccination and Infection. J. Med. Virol..

[13] Teruel N.F.B., Crown M., Rajsbaum R., Bashton M., Najmanovich R. (2025). Comprehensive Analysis of SARS-CoV-2 Spike Evolution: Epitope Classification and Immune Escape Prediction. Virus Evol..

[14] Dissanayake U.C., Roy A., Maghsoud Y., Polara S., Debnath T., Cisneros G.A. (2025). Computational Studies on the Functional and Structural Impact of Pathogenic Mutations in Enzymes. Protein Sci..

[15] Akbulut E. (2022). Investigation of Changes in Protein Stability and Substrate Affinity of 3CL-Protease of SARS-CoV-2 Caused by Mutations. Genet. Mol. Biol..

[16] Suleman M., Murtaza A., Maria, Khan H., Rashid F., Alshammari A., Ali L., Khan A., Wei D.-Q. (2023). The XBB. 1.5 Slightly Increase the Binding Affinity for Host Receptor ACE2 and Exhibit Strongest Immune Escaping Features: Molecular Modeling and Free Energy Calculation. Front. Mol. Biosci..

[17] Bosch B.J., van der Zee R., de Haan C.A.M., Rottier P.J.M. (2003). The Coronavirus Spike Protein Is a Class I Virus Fusion Protein: Structural and Functional Characterization of the Fusion Core Complex. J. Virol..

[18] Hoffmann M., Kleine-Weber H., Schroeder S., Krüger N., Herrler T., Erichsen S., Schiergens T.S., Herrler G., Wu N.H., Nitsche A. (2020). SARS-CoV-2 Cell Entry Depends on ACE2 and TMPRSS2 and Is Blocked by a Clinically Proven Protease Inhibitor. Cell.

[19] Walls A.C., Tortorici M.A., Snijder J., Xiong X., Bosch B.J., Rey F.A., Veesler D. (2017). Tectonic Conformational Changes of a Coronavirus Spike Glycoprotein Promote Membrane Fusion. Proc. Natl. Acad. Sci. USA.

[20] Popovic M.E. (2023). XBB. 1.5 Kraken Cracked: Gibbs Energies of Binding and Biosynthesis of the XBB. 1.5 Variant of SARS-CoV-2. Microbiol. Res..

[21] Parums D.V. (2023). The Xbb. 1.5 (‘Kraken’) Subvariant of Omicron Sars-Cov-2 and Its Rapid Global Spread. Med. Sci. Monit..

[22] Mannar D., Saville J.W., Zhu X., Srivastava S.S., Berezuk A.M., Tuttle K.S., Marquez A.C., Sekirov I., Subramaniam S. (2022). SARS-CoV-2 Omicron Variant: Antibody Evasion and Cryo-EM Structure of Spike Protein–ACE2 Complex. Science.

[23] Kumar S., Karuppanan K., Subramaniam G. (2022). Omicron (BA. 1) and Sub-variants (BA. 1.1, BA. 2, and BA. 3) of SARS-CoV-2 Spike Infectivity and Pathogenicity: A Comparative Sequence and Structural-based Computational Assessment. J. Med. Virol..

[24] Sharma T., Gerstman B., Chapagain P. (2023). Distinctive Features of the XBB. 1.5 and XBB. 1.16 Spike Protein Receptor-Binding Domains and Their Roles in Conformational Changes and Angiotensin-Converting Enzyme 2 Binding. Int. J. Mol. Sci..

[25] Wang X., Jiang S., Jiang S., Li X., Ai J., Lin K., Lv S., Zhang S., Li M., Li J. (2023). Neutralization of SARS-CoV-2 BQ. 1.1, CH. 1.1, and XBB. 1.5 by Breakthrough Infection Sera from Previous and Recent Waves in China. Cell Discov..

[26] Liu S., Liang Z., Nie J., Gao W.B., Li X., Zhang L., Yu Y., Wang Y., Huang W. (2023). Sera from Breakthrough Infections with SARS-CoV-2 BA. 5 or BF. 7 Showed Lower Neutralization Activity against XBB. 1.5 and CH. 1.1. Emerg. Microbes Infect..

[27] Ghoula M., Deyawe Kongmeneck A., Eid R., Camproux A.-C., Moroy G. (2023). Comparative Study of the Mutations Observed in the SARS-CoV-2 RBD Variants of Concern and Their Impact on the Interaction with the ACE2 Protein. J. Phys. Chem. B.

[28] Verkhivker G., Alshahrani M., Gupta G. (2023). Balancing Functional Tradeoffs between Protein Stability and ACE2 Binding in the SARS-CoV-2 Omicron BA. 2, BA. 2.75 and XBB Lineages: Dynamics-Based Network Models Reveal Epistatic Effects Modulating Compensatory Dynamic and Energetic Changes. Viruses.

[29] Al Khalaf R., Bernasconi A., Pinoli P. (2024). Systematic Analysis of SARS-CoV-2 Omicron Subvariants’ Impact on B and T Cell Epitopes. PLoS ONE.

[30] Vita R., Blazeska N., Marrama D., Duesing S., Bennett J., Greenbaum J., Mendes M.D.A., Mahita J., Wheeler D.K., Cantrell J.R. (2025). The Immune Epitope Database (IEDB): 2024 Update. Nucleic Acids Res..

[31] Mengist H.M., Kombe A.J.K., Mekonnen D., Abebaw A., Getachew M., Jin T. (2021). Mutations of SARS-CoV-2 Spike Protein: Implications on Immune Evasion and Vaccine-Induced Immunity. Semin. Immunol..

[32] Singh J., Pandit P., McArthur A.G., Banerjee A., Mossman K. (2021). Evolutionary Trajectory of SARS-CoV-2 and Emerging Variants. Virol. J..

[33] Khan M.S., Shakya M., Verma C.K., Mukherjee R. (2024). Identification of Highly Conserved Surface-Exposed Peptides of Spike Protein for Multiepitope Vaccine Design against Emerging Omicron Variants: An Immunoinformatic Approach. Hum. Immunol..

[34] Mazzoni A., Vanni A., Spinicci M., Capone M., Lamacchia G., Salvati L., Coppi M., Antonelli A., Carnasciali A., Farahvachi P. (2022). SARS-CoV-2 Spike-Specific CD4+ T Cell Response Is Conserved against Variants of Concern, Including Omicron. Front. Immunol..

[35] Rao X., Zhao R., Tong Z., Guo S., Peng W., Liu K., Li S., Wu L., Tong J., Chai Y. (2023). Defining a de Novo Non-RBM Antibody as RBD-8 and Its Synergistic Rescue of Immune-Evaded Antibodies to Neutralize Omicron SARS-CoV-2. Proc. Natl. Acad. Sci. USA.

[36] Wang Q., Guo Y., Liu L., Schwanz L.T., Li Z., Nair M.S., Ho J., Zhang R.M., Iketani S., Yu J. (2023). Antigenicity and Receptor Affinity of SARS-CoV-2 BA. 2.86 Spike. Nature.

[37] He C., Alu A., Lei H., Yang J., Hong W., Song X., Li J., Yang L., Wang W., Shen G. (2023). A Recombinant Spike-XBB. 1.5 Protein Vaccine Induces Broad-spectrum Immune Responses against XBB. 1.5-included Omicron Variants of SARS-CoV-2. MedComm.

[38] Ishimaru H., Nishimura M., Shigematsu H., Marini M.I., Hasegawa N., Takamiya R., Iwata S., Mori Y. (2024). Epitopes of an Antibody That Neutralizes a Wide Range of SARS-CoV-2 Variants in a Conserved Subdomain 1 of the Spike Protein. J. Virol..

[39] Lauster D., Haag R., Ballauff M., Herrmann A. (2025). Balancing Stability and Function: Impact of the Surface Charge of SARS-CoV-2 Omicron Spike Protein. npj Viruses.

[40] Dadonaite B., Brown J., McMahon T.E., Farrell A.G., Figgins M.D., Asarnow D., Stewart C., Lee J., Logue J., Bedford T. (2024). Spike Deep Mutational Scanning Helps Predict Success of SARS-CoV-2 Clades. Nature.

[41] Park S.B., Khan M., Chiliveri S.C., Hu X., Irvin P., Leek M., Grieshaber A., Hu Z., Jang E.S., Bax A. (2023). SARS-CoV-2 Omicron Variants Harbor Spike Protein Mutations Responsible for Their Attenuated Fusogenic Phenotype. Commun. Biol..

[42] Melero R., Sorzano C.O.S., Foster B., Vilas J.-L., Martínez M., Marabini R., Ramírez-Aportela E., Sanchez-Garcia R., Herreros D., del Cano L. (2020). Continuous Flexibility Analysis of SARS-CoV-2 Spike Prefusion Structures. IUCrJ.

[43] Akbulut E. (2021). SARS CoV-2 Spike Glycoprotein Mutations and Changes in Protein Structure. Trak. Univ. J. Nat. Sci..

[44] NCBI NCBI Virus. https://www.ncbi.nlm.nih.gov/labs/virus/vssi/#/.

[45] Katoh K., Rozewicki J., Yamada K.D. (2018). MAFFT Online Service: Multiple Sequence Alignment, Interactive Sequence Choice and Visualization. Brief. Bioinform..

[46] Tamura K., Stecher G., Kumar S. (2021). MEGA11: Molecular Evolutionary Genetics Analysis Version 11. Mol. Biol. Evol..

[47] Waterhouse A., Bertoni M., Bienert S., Studer G., Tauriello G., Gumienny R., Heer F.T., De Beer T.A.P., Rempfer C., Bordoli L. (2018). Swiss-Model: Homology Modelling of Protein Structures and Complexes. Nucleic Acids Res..

[48] Camacho C., Coulouris G., Avagyan V., Ma N., Papadopoulos J., Bealer K., Madden T.L. (2009). BLAST+: Architecture and Applications. BMC Bioinform..

[49] Remmert M., Biegert A., Hauser A., Söding J. (2012). HHblits: Lightning-Fast Iterative Protein Sequence Searching by HMM-HMM Alignment. Nat. Methods.

[50] Walls A.C., Park Y.J., Tortorici M.A., Wall A., McGuire A.T., Veesler D. (2020). Structure, Function, and Antigenicity of the SARS-CoV-2 Spike Glycoprotein. Cell.

[51] Studer G., Tauriello G., Bienert S., Biasini M., Johner N., Schwede T. (2021). ProMod3—A Versatile Homology Modelling Toolbox. PLoS Comput. Biol..

[52] Bertoni M., Kiefer F., Biasini M., Bordoli L., Schwede T. (2017). Modeling Protein Quaternary Structure of Homo- and Hetero-Oligomers beyond Binary Interactions by Homology. Sci. Rep..

[53] Baek M., DiMaio F., Anishchenko I., Dauparas J., Ovchinnikov S., Lee G.R., Wang J., Cong Q., Kinch L.N., Dustin Schaeffer R. (2021). Accurate Prediction of Protein Structures and Interactions Using a Three-Track Neural Network. Science.

[54] Benkert P., Tosatto S.C.E., Schomburg D. (2008). QMEAN: A Comprehensive Scoring Function for Model Quality Assessment. Proteins: Struct. Funct. Bioinform..

[55] Jespersen M.C., Peters B., Nielsen M., Marcatili P. (2017). BepiPred-2.0: Improving Sequence-Based B-Cell Epitope Prediction Using Conformational Epitopes. Nucleic Acids Res..

[56] Emini E.A., Hughes J.V., Perlow D., Boger J. (1985). Induction of Hepatitis A Virus-Neutralizing Antibody by a Virus-Specific Synthetic Peptide. J. Virol..

[57] Kolaskar A.S., Tongaonkar P.C. (1990). A Semi-Empirical Method for Prediction of Antigenic Determinants on Protein Antigens. FEBS Lett..

[58] Pires D.E.V., Ascher D.B., Blundell T.L. (2014). MCSM: Predicting the Effects of Mutations in Proteins Using Graph-Based Signatures. Bioinformatics.

[59] Zhou Y., Pan Q., Pires D.E.V., Rodrigues C.H.M., Ascher D.B. (2023). DDMut: Predicting Effects of Mutations on Protein Stability Using Deep Learning. Nucleic Acids Res..

[60] Pires D.E.V., Ascher D.B., Blundell T.L. (2014). DUET: A Server for Predicting Effects of Mutations on Protein Stability Using an Integrated Computational Approach. Nucleic Acids Res..

[61] Rodrigues C.H.M., Pires D.E.V., Ascher D.B. (2021). DynaMut2: Assessing Changes in Stability and Flexibility upon Single and Multiple Point Missense Mutations. Protein Sci..

[62] Choi Y., Chan A.P. (2015). PROVEAN Web Server: A Tool to Predict the Functional Effect of Amino Acid Substitutions and Indels. Bioinformatics.

[63] Sim N.-L., Kumar P., Hu J., Henikoff S., Schneider G., Ng P.C. (2012). SIFT Web Server: Predicting Effects of Amino Acid Substitutions on Proteins. Nucleic Acids Res..

[64] Adzhubei I., Jordan D.M., Sunyaev S.R. (2013). Predicting Functional Effect of Human Missense Mutations Using PolyPhen-2. Curr. Protoc. Hum. Genet..

[65] Li H., Chang Y.Y., Lee J.Y., Bahar I., Yang L.W. (2017). DynOmics: Dynamics of Structural Proteome and Beyond. Nucleic Acids Res..

[66] Chawla M., Kalra U., Petta A., Sharma S., Shaikh A.R., Cavallo L., Oliva R. (2025). COCOMAPS 2.0: A Web Server for Identifying, Analyzing, and Visualizing Atomic Interactions at the Interface of Biomolecular Complexes. Bioinformatics.

